# Does pre-testing promote better retention than post-testing?

**DOI:** 10.1038/s41539-019-0053-1

**Published:** 2019-09-24

**Authors:** Alice Latimier, Arnaud Riegert, Hugo Peyre, Son Thierry Ly, Roberto Casati, Franck Ramus

**Affiliations:** 1 Laboratoire de Sciences Cognitives et Psycholinguistique, Département d’Etudes Cognitives, ENS, EHESS, CNRS, PSL University, Paris, France; 2 Institut Jean Nicod, ENS, EHESS, CNRS, PSL University, Paris, France; 3 Didask, Paris, France; 4 Université Paris Diderot, Sorbonne Paris Cité, UMRS 1141, Paris, France; 5 Assistance Publique-Hôpitaux de Paris, Robert Debré Hospital, Child and Adolescent Psychiatry Department, Paris, France

**Keywords:** Human behaviour, Education

## Abstract

Compared with other learning strategies, retrieval practice seems to promote superior long-term retention. This has been found mostly in conditions where learners take tests after being exposed to learning content. However, a pre-testing effect has also been demonstrated, with promising results. This raises the question, for a given amount of time dedicated to retrieval practice, whether learners should be tested before or after an initial exposure to learning content. Our experiment directly compares the benefits of post-testing and pre-testing relative to an extended reading condition, on a retention test 7 days later. We replicated both post-testing (*d* = 0.74) and pre-testing effects (*d* = 0.35), with significantly better retention in the former condition. Post-testing also promoted knowledge transfer to previously untested questions, whereas pre-testing did not. Our results thus suggest that it may be more fruitful to test students after than before exposure to learning content.

## Introduction

The testing effect is a strong and well demonstrated effect.^1–5^ As opposed to common learning practices such as reading, taking tests, and more generally retrieval practice during the learning phase contribute to better long term retention^6^ by reducing the forgetting rate of information across time.^7^ The benefits of retrieval practice have been demonstrated in both laboratory and classroom settings^8,9^ and for both simple (e.g. word lists) and complex (e.g. prose passages) material.^10^ In a meta-analysis, Rowland^11^ reported a mean effect size of *g* = 0.50 [IC-95%: 0.42, 0.58] from 61 studies comparing the effects of testing vs. restudying on the ability to learn new information after a first exposure to learning contents. In another meta-analysis, Adesope et al.^12^ found a mean effect size of *g* = 0.61 [IC-95%: 0.58 and 0.65] by comparing retrieval practice to other practices.

The testing effect may also lead to better retention of previously untested information and to greater knowledge transfer than restudying.^13–15^ A recent meta-analysis^16^ on the transfer of retrieval practice effects found that retrieval practice yielded transferrable learning relative to a restudying control condition (*d* = 0.40, 95%CI [0.31 and 0.50]). However, transfer does not necessarily occur in all circumstances and with all types of content.^14^ Pan and Rickard^16^ made a distinction between untested application and inference questions and untested information seen during the first exposure to material. Interestingly, they found a transfer effect of retrieval practice on application and inference questions, but not on untested information seen during initial study.

The testing effect has mainly been shown in the context of tests given after exposure to learning contents. However, a pre-testing effect has also been shown in laboratory settings with promising results. Indeed, taking a test before being exposed to learning content enhances retention compared with no retrieval practice.^17–23^ Although the pre-testing effect was demonstrated on written materials (prose passages as well as paired words), a recent study also found it on video-based learning as well.^17^ Moreover, Hartley’s results indicated that the harder the pre-tested questions, the larger the improvement on retention. Even when the rate of success obtained in the pre-test was very low (in Richland et al.’s study, participants got as many as 95% of the pre-test answers wrong), learning with a pre-test was better than just studying twice the content.^22,24^ Some of these studies reported that pre-testing also facilitated the learning of untested information,^17,19,25^ while others found an effect only for pre-tested information, suggesting a lack of transfer.^21,22^ In two studies, there was actually a decrease in final performance for the untested information compared with a control group with no pre-test, suggesting a detrimental effect on untested material.^20,23^ This was also found in a literature review on question position when learning prose materials.^26^ Thus, while the post-testing effect seems to transfer to untested material under certain conditions,^16^ the evidence is more ambivalent for the pre-testing effect. One might predict that pre-testing might narrow attentional focus to tested information only, thus harming the learning of untested information.

In terms of putative mechanisms, the experiments of Pressley et al.^21^ and Richland et al.^22^ showed that just reading the pre-test is insufficient to enhance final retention, it is the process of attempting to find relevant answers that accounts for the pre-testing effect. Generating an answer, even an incorrect one, may strengthen the retrieval routes between the question and the correct answer and encourage deep processing.^24^ Furthermore, being exposed to questions ahead of time may also help focus one’s attention to the most relevant parts of the learning content. However, these studies showed an effect of pre-testing on retention only when compared with additional study, or to a passive-learning condition.

Given prior knowledge on the testing effect, and in a context of trying to optimise the time allocated to learning, a more relevant question would be: Should teachers test their students before or after the lecture? Existing studies have only tangentially addressed the question. Studies conducted by Frase^27,28^ were not exactly a comparison of pre- and post-testing, but compared placing adjunct questions within prose passages either before or after target information, and found better retention in the latter case. Similarly, in Sagaria and Di Vesta’s^23^ study, participants read pairs of paragraphs and questions, and the authors compared the effects of reading and copying each question just before or just after reading the paragraph. Thus this experimental situation does not really emulate testing before or after a lecture. Two quantitative reviews of such studies on adjunct questions during learning of prose materials reported similar effects of pre- and post-target information questions on tested material, but an advantage of post-questions on untested material.^26,29^ However, the studies did not directly compare the effect of questions before and after reading a prose passage. Rather, pre- and post-testing effects measured in different studies were compared. Finally, McDaniel et al.’s^30^ Experiments 2A and 2B compared the effects of pre- and post-lesson quizzes, but in fact both quizzes and the lecture were preceded by an initial reading of the chapter, so this did not strictly speaking constitute pre-testing but rather interim testing. Both experiments reported similar results, i.e., lower final performance in the pre-test than in the post-test condition.

In 2007, the US Department of Education published a summary report with several recommendations for improving teaching to reinforce learning.^31^ One of them was to “use pre-questions to introduce a new topic”. Yet the level of evidence associated with this recommendation was indicated to be low, because of the scarcity of relevant studies on the pre-testing effect. However, in the 12 years since the publication of the practice guide, the potential of pre-testing to promote better learning in educational settings still remains little explored. It therefore remains important to measure in the same experiment whether it is truly beneficial to spend time testing students before the first exposure to learning content, rather than afterwards. Furthermore, it is also uncertain whether pre-testing promotes as much transfer to untested material as post-testing does.

In this paper, we report an experiment that directly compares the learning effects of pre-testing (quiz-reading condition), post-testing (reading-quiz condition), and re-reading (reading-reading condition) on long term memory. Specifically, we aimed to determine (i) the size of testing effects, and how they compare between pre-testing and post-testing; (ii) to what extent pre- and post-testing benefits transfer to questions that were not tested (trained vs. untrained questions).

## Results

### Participants’ data

From a total of 334 recruited participants, 44 participants (13%) gave up participation between the learning phase and the final test, four participants were excluded because they did not complete the learning phase entirely, and one was excluded for completing the learning phase twice in a day, so we had a total of 285 participants that were included in the analysis. Demographic data are indicated in Table 1. An independent-samples *t*-test revealed no significant differences between the three groups in terms of age and education level (ps > 0.05). The sex ratio did not differ either (*χ*^2^ (2) = 0.32 and *p* = 0.85). We also asked participants to estimate their degree of knowledge on DNA on a scale from 1 (“I do not have any knowledge on DNA”) to 5 (“I have extensive knowledge on DNA”). Most of the participants were unfamiliar with DNA (*M* = 1.59; S.D = 0.69), and this did not differ between the groups (ps > 0.5).

**Table 1:** Summary characteristics of the three groups of participants (reading-reading, quiz-reading, and reading-quiz)

	Learning conditions			
	Reading-reading	Quiz-reading	Reading-quiz	Total
*N*	95	95	95	285
Gender (male/female)	40/55	39/56	36/59	115/170
Age (years)	34.8 (9)	35.9 (9)	37.7 (10.9)	36.1 (9.7)
Education (years)	14.4 (1.7)	14.6 (1.7)	14.5 (1.9)	14.6 (1.9)
Degree of knowledge on DNA (from 1 to 5)	1.6 (0.7)	1.6 (0.7)	1.6 (0.7)	1.6 (0.7)

Standard deviations are in parentheses

### Training phase

#### Quiz performance

Even though we instructed participants to complete each training module only once, a few participants returned to some of the modules a second time. The mean number of module studied (for a total of 7) during the training phase was 7.24 (*SD* = 0.86) in the quiz-reading group and 7.56 (*SD* = 1.13) in the reading-quiz group (*t*(183) = 2.14, *p* = 0.033, and *d* = 0.31). This variable was used as a covariate for the final test analyses. When taking into account only the first attempt for each question, the reading-quiz group had a better total score (*M* = 68.09%, *SD* = 16.99%) than the quiz-reading group (*M* = 47.15%, *SD* = 12.92%), *t*(188) = 9.56, *p* < 0.0001, and *d* *=* 1.39.

#### Training times

We also computed the total time spent in the training phase, i.e. the cumulative time spent on learning contents and on quizzes, including quizzes that were taken twice or more than once. For technical reason, training time could not be calculated for eight participants.

Because times are not normally distributed, statistics were computed on the natural logarithm of the total training time. There was a main effect of the learning condition on the time spent on training contents, F(2,274) = 125.52, *p* < 0.0001, and *η*^2^ = 0.48. The amount of time spent on the training session was longer for the quiz-reading group than for the reading-quiz group. Both the groups with quizzes spent significantly more time on the training session than the group that was not assigned to learn with quizzes (Table 2).

**Table 2:** Time spent on the training in log(min) and effect sizes for the planned *t*-test comparisons between the different groups of participants

Learning conditions			Planned *t*-test comparisons		
Reading-reading	Reading-quiz	Quiz-reading	Reading-quiz vs.	Quiz-reading vs.	Quiz-reading vs.
			Reading-reading	Reading-quiz	Reading-reading
2.87	3.59	3.76	*d* = 1.94 [1.59, 2.29]	*d* = 0.35 [0.05, 0.64]	*d* = 1.89 [1.54, 2.24]
(0.35)	(0.40)	(0.47)	*p* < 0.0001	*p* = 0.011	*p* = 0.013

Standard deviations are in parentheses. Effect sizes (Cohen’s *d*) are given with 95% confidence intervals

### Final test phase

A two-way ANOVA showed a main effect of the learning condition, F(2,564) = 13.56, *p* < 0.0001, and *η*^2^ = 0.035; with the reading-quiz group showing the best final performance, then the quiz-reading group, and finally the reading-reading group (Table 3). There was also a main effect of question type (F(1,564) = 187.78, *p* < 0.0001, and *η*^2^ = 0.24). The questions trained during the learning phase were more successfully answered by participants (*M* = 59.51% and *SD* = 19.57%) than the untrained questions (*M* = 39.06% and *SD* = 16.84%). The correlation between the two types of questions was *r* = 0.71 and *p* < 0.0001. There was a trend for an interaction between the learning condition and question type variables (F(2,564) = 2.70, *p* = 0.068, and *η*^2^ = 0.007).

**Table 3:** Final performance for each learning condition (reading-reading, reading-quiz, and quiz-reading) to the trained and untrained questions (new, generalisation, and total untrained) and across questions

Learning conditions			
	Reading-reading	Reading-quiz	Quiz-reading
Trained questions	52.87% (17.94%)	66.15% (19.94%)	59.51% (18.79%)
New questions	39.04% (17.44%)	43.73% (20.64%)	37.13% (16.86%)
Generalisation questions	35.37% (18.44%)	41.68% (21.72%)	37.16% (20.19%)
Untrained questions (total)	37.29% (15.2%)	42.76% (18.61%)	37.14% (16.08%)
All questions	45.91% (15.82%)	55.70% (17.88%)	49.52% (16.03%)

Standard deviations are in parentheses

**Fig. 1:**
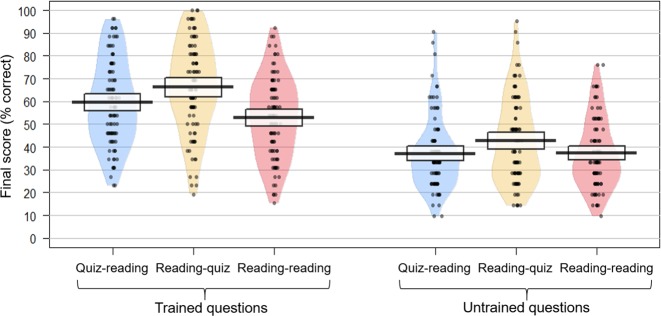
Final test performance to the trained and untrained questions (percentage correct answers) and for each group of participants. Each point is a participant. The thick horizontal line represents the median, colour-shaded bean plots show the full distribution of the data, and boxes represent 95% Confidence Interval

To answer our research questions, we conducted separate one-way ANOVA analyses for trained, new, and generalisation questions as dependent variable, and the three learning conditions as factor. Furthermore, considering the relatively low number of new and generalisation questions, and the absence of significant difference between them, we also grouped them in a post-hoc analysis of the category “untrained questions”. Thus, the two dependent variables—trained and untrained question scores—were analysed as the within-subject variable Question Type. Tables 3 and 4 and Fig. 1 summarise final recall for each question type (trained and untrained) and learning condition, and post-hoc analysis for each one-way ANOVA.

**Table 4:** Effect sizes for the planned *t*-test comparisons between the different conditions (the post-testing effect, the effect of quiz position, and the pre-testing effect) to the trained and untrained questions (new, generalisation, and total untrained) and across questions

	Planned *t*-test comparisons		
	Reading-quiz vs.	Reading-quiz vs.	Quiz-reading vs.
	Reading-reading	Quiz-reading	Reading-reading
Trained questions	*d* = 0.74 [0.44, 1.04]	*d* = 0.34 [0.05, 0.63]	*d* = 0.35 [0.07, 0.64]
	*p* < 0.0001	*p* = 0.019	*p* = 0.013
New questions	*d* = 0.27 [−0.02, 0.56]	*d* = 0.35 [0.06, 0.64]	*d* = −0.11 [−0.40, 0.17]
	*p* *=* 0.092	*p* = 0.017	*p* = 0.44
Generalisation questions	*d* = 0.34 [0.05, 0.63]	*d* = 0.22 [−0.07, 0.50]	*d* = 0.09 [−0.20, 0.37]
	*p* = 0.032	*p* = 0.14	*p* = 0.52
Untrained questions (total)	*d* = 0.36 [0.07, 0.66]	*d* = 0.32 [0.03, 0.61]	*d* = −0.01 [−0.29, 0.27]
	*p* = 0.028	*p* = 0.027	*p* = 0.95
All questions	*d* = 0.62 [0.32, 0.92]	*d* = 0.36 [0.07, 0.65]	*d* = 0.22 [−0.06, 0.51]
	*p* < 0.0001	*p* = 0.013	*p* = 0.12

Effect sizes (Cohen’s *d*) are given with 95% confidence intervals

#### Trained questions

Consistent with predictions, there was a main effect of the learning condition, F(2,282) = 11.76, *p* < 0.0001, and *η*^*2*^ = 0.077. As shown in Table 4, planned *t*-test comparisons showed a post-testing effect, a pre-testing effect, and most interestingly, a position effect: retention was significantly higher for the reading-quiz group compared with the quiz-reading group (Table 3).

#### Untrained “new” questions

There was a main effect of the learning condition, F(2,282) = 3.24, *p* = 0.041, and *ηp*^2^ = 0.022. Planned *t*-test comparisons did not show a significant post-testing effect or a pre-testing effect (Table 4). However, there was a significant position effect: the reading-quiz group had better final performance than the quiz-reading group (Table 3).

#### Untrained “generalisation” questions

No main effect of the learning condition was found, F(2,282) = 2,48, *p* = 0.086, and *ηp*^2^ = 0.017. For information, effect sizes for planned *t*-test comparisons are presented in Table 4.

#### Untrained questions (new and generalisation)

There was a main effect of the learning condition, F(2,282) = 3.49, *p* = 0.032, and *η*^2^ = 0.024. As shown in Tables 3 and 4, comparison of final test performance on this question type revealed a significant advantage for the reading-quiz group on both reading-reading (post-testing effect) and quiz-reading groups (position effect). However, final test performance for the untrained information in the quiz-reading group was not different from the performance in the reading-reading group (no pre-testing effect).

### Covariates and exploratory analyses

In the multiple linear regression model analysis restricted to the two learning conditions with quizzes, we added the total number of attempts to take the quizzes and the log of the time spent on training as covariates. The total number of attempts to take the quizzes had an influence on the final test score, F(1,352) = 4.82, *p* = 0.03, and *η*^2^ = 0.013. However, the influence of the log(training time) was not significant, F(1,352) = 2.01, *p* = 0.157372, and *η*^2^ < 0.01. Main effects of the learning condition (reading-quiz vs. quiz-reading) and of the question type (trained vs. untrained) were still significant (F(1,352) = 8.86, *p* < 0.01, and *η*^2^ = 0.014; and F(1,352) = 140.83, *p* < 0.0001, and *η*^2^ = 0.28), but not the interaction F(1,352) = 0.10, *p* = 0.75, and *η*^2^ < 0.001.

Then, in the full multiple linear regression model (three learning conditions × two question types), adding log(training time) had no significant effect (F(1,547) = 0.33, *p* = 0.56, and *η*^2^ < 0.001). The main effects of the learning condition and of the question type (trained vs. untrained) remained significant (F (2,547) = 13.63, *p* < 0.0001, and *η*^2^ = 0.02; and F (1,547) = 180.43, *p* < 0.0001, and *η*^2^ = 0.24). There was a trend for a significant interaction (F(2.547) = 2.76, *p* = 0.06, and *ηp*^2^ < 0.01).

Finally, and as an exploratory analysis, we added the participants’ age and degree of knowledge about DNA as covariates in the full linear model too. Age had a significant influence on final test score such as when age increased, final test performances tended to decrease (F(1,460) = 8.94, *p* *<* 0.01, and *η*^2^ < 0.01); as expected, the degree of knowledge about DNA had a significant positive effect (F(1,460) = 28.05, *p* *<* 0.01, and *η*^2^ = 0.04). Like in the initial analyses, the learning condition and question type factors remained significant (F (2,460) = 13.21, *p* < 0.0001, and *η*^2^ = 0.05; and F(1,460) = 153.37, *p* < 0.0001, and *η*^2^ = 0.23). The interaction was not significant, F (2,460) = 2.21, *p* = 0.11, and *η*^2^ < 0.01.

## Discussion

The results from this experiment provide insights into the effective placement of retrieval practice relative to studying the content as well as replicate results from previous research with robust data. First, significant testing effects (in comparison to reading-reading time) were found when quizzes were placed either after (post-testing effect) or before reading the same contents (pre-testing effect), in line with previous findings.^26,29^ Furthermore, the post-testing effect was significantly larger than the pre-testing effect. Finally, whereas post-testing increased the retention of related but untrained material, pre-testing did not. These results were obtained for the learning of prose passages of scientific content, in a digital learning environment, and with a retention interval of 7 days.

Regarding the post-testing effect, we replicated the same large effect size (*d* = 0.62 across question types) as found by Rowland^11^ in his meta-analysis of between-subject experiments (*g* = 0.69). This effect was particularly strong for the trained questions (*d* = 0.74), and smaller but significant for the untrained questions (*d* = 0.32). Thus, initial testing led to enhanced recall for untrained but related questions. This latter result is consistent with that of Chan et al.,^14^ although with a smaller effect size (in their Experiment 1, they obtained *d* = 0.69 for the testing group on untested questions relative to the reading-reading group, and *d* = 0.56 relative to the control group). The benefit of post-testing practice on untrained questions seems to be driven by generalisation rather than new questions (whose answer was present in the learning material). This is in line with a recent meta-analysis on the transfer of learning:^16^ the authors found no evidence for transfer of the testing effect to untested materials seen during initial study (similar to our new question type), but they found an overall positive transfer on application and inference questions (similar to our generalisation question type).

Regarding the pre-testing effect, we found a smaller but significant effect on trained questions (*d* = 0.35) but not on untrained questions (*d* = −0.01). This is rather lower than in previous recent studies. In Richland et al.’s study,^22^ participants had a very low success rate in their pre-test sessions (around 5%), which provided a huge margin of improvement. In contrast, in our experiments, the mean percentage correct during the learning phase was 40% in the quiz-reading condition, which may indicate a certain level of prior knowledge, and provides less margin of improvement (although there was no hint of a ceiling effect in the final test).

Our study replicates previous results with educational contents and huge sample size. These results are consistent not only with those of Frase^27,28^ showing an advantage of post-test over pre-test location but also with previous studies that compared post-testing and combined pre- and post-testing and found little difference.^30^ Similarly, a new study by Geller et al.^32^ found that asking questions before having a lecture did not enhance the learning of the pre-tested information more than the learning of other information which was not pre-tested. Moreover, doing a pre-test did not boost the benefits of the post-testing effect.

What may account for the superiority of post-testing over pre-testing? First, unlike pre-testing, post-testing enables the consolidation of previously read information. Second, in the quiz-reading condition, participants answered incorrectly and therefore received negative feedback much more frequently than in the reading-quiz condition. This decreased reward may have affected their motivation and learning, although the notion of “desirable difficulties” would have predicted the opposite.^33^ Third, and in relation to this second point, participants in the quiz-reading condition had on average lower initial performance during the training phase in comparison to the reading-quiz participants. Rowland^11^ found that higher initial performance may increase the magnitude of the testing effect. Thus, the difference in initial training performance between reading-quiz and quiz-reading groups might explain all or part of the performance difference in the final test. However, exploratory covariance analyses adjusting for initial test performance showed that the main group effect remained the same irrespective of initial test performance.

Interestingly, we observed no difference between the quiz-reading and the reading-reading condition on untrained questions (neither for new nor for generalisation questions), consistently with previous research.^21,22^ Moreover, performance on new questions was significantly greater for post- than for pre-testing. One possible explanation is that, during the reading phase, participants in the post-testing condition were not induced to prioritise among the available information: all pieces of information were a priori equally relevant and therefore perhaps equally attended. In the pre-testing condition, however, participants may have been incited to focus their attention on the answers to pre-tested questions, to the detriment of other material that was used to create the new untrained questions for the final test. By contrast, Carpenter and Toftness^17^ found a benefit of pre-testing on both tested and untested information but in the context of video-based learning. Thus, the effect of pre-testing on the learning of untested information might be dependent of the format of the learning content, and may be harmful to learning by encouraging selective attention when material is read but not listened.

A potential limitation of the present study might be that trained and untrained questions were not counterbalanced across participants. Thus, the greater performance on trained questions may be due both to training, and to the fact that untrained questions were intrinsically more difficult (as suggested by the lower performance on untrained questions even in the reading-reading condition). This would be a problem if our main goal was to compare absolute performance between trained and untrained questions. However, our interest here was rather to investigate the interaction between question familiarity and test location.

Our conclusions are also a priori limited to a certain type of material (scientific text), mode of presentation (an e-learning platform), and to a certain type of participants, i.e., workers doing a paid task, rather than students. It will be important to address the same question using other learning contents and populations, especially school children in a more ecological context. Nevertheless, our replication of the well-established post-testing, pre-testing, and transfer effects suggests that our experimental conditions produce similar results as other studies in different conditions, so we see no reason to suspect that our new result (the superiority of post- over pre-testing) would not generalise.

Contrary to previous experiments where training time was closely matched across conditions,^2,22^ in the present experiment participants were free to spend as much time as they felt necessary on the contents (prose passages, quizzes, and feedback). This induced important differences in training time between conditions, with participants in the reading-reading condition spending on average less than half the time in training than participants in the two quiz conditions, and participants in the quiz-reading condition spending 18% more time on training than those in the reading-quiz condition. However, we found no correlation whatsoever between training time and final performance, and covariance analyses adjusting for training time obtained exactly the same results. Thus, our pre- and post-testing effects cannot be attributed to the extra time spent on training, and the extra time spent on pre- vs. post-testing would predict the opposite from the observed advantage of post-testing.

Finally, we were unable to exclude or control potential extra study time between the training phase and the final test. However, participants were paid for participation regardless of performance, thus they had no incentive to spend more time studying than the minimum imposed. Furthermore, there is no reason to think that participants in different groups might have invested differently in extra study. Therefore, there is no reason to think that this may have changed the pattern of results.

To conclude, our results may help refine the recommendation of the US Department of Education^31^ about the use of pre-questions to foster learning in classroom settings. It is important to note that pre-questions may be used in different ways and serve different functions. Even though pre-testing did not enhance retention as much as post-testing in our experiment, there may be other benefits of asking questions before a lecture. For instance, pre-questions may be used to test whether prerequisite knowledge is acquired, and to refresh it right before new content is exposed. In that case, those so-called pre-questions actually implement post-testing of previously learned material. However, our results do not support the specific idea that pre-questions on the content of the subsequent lecture improve learning more than post-questions. Thus, current evidence suggests that testing time dedicated to enhancing retention is better spent after than before the initial exposure to learning content.

## Methods

### Participants

We calculated that at least 64 participants per group were necessary in order to detect a testing effect of size *d* = 0.50^11^ in a between-subjects design with a statistical power of 0.8 (alpha = 0.05, bilateral *t*-test). Because we anticipated that the pre-testing effect might be smaller than the post-testing effect, and because we wanted to compare the pre- and post-testing effects, we aimed at including 100 participants per group, thus giving us 80% power to detect an effect size of 0.4. We therefore recruited a total of 334 adult participants via a French online work platform (Foule Factory https://www.foulefactory.com/). Inclusion criteria were that participants are native French speakers and without any reported neurological or psychiatric disorders. Furthermore, participation of students and professionals in medicine or biology was discouraged in the call for volunteers and in the information letter. All participants provided written informed consent on the online platform. The study was approved by the local research ethics committee (Comité d’Ethique de la Recherche of Paris Descartes University) and the participants were paid for their participation.

### Material

The experiment was entirely run online on the Didask digital learning platform (https://www.didask.com/). On Didask, each course consists of a set of modules organised in a logical order. A module is an elementary learning unit, including both learning material (text, videos, pictures, …) and a corresponding training quiz with at least five questions (multiple choice, pairwise matching, ordering, sorting into categories, …) and can last between 5 and 15 min. We used a modified version of Didask designed for experimental testing, allowing us to specify several learning conditions and to better control the learning environment, in particular the order in which the content and the quiz were presented.

Study material consisted of a course on DNA from the French curriculum for 12th grade science tracks (age range: 18 years old). Specific contents were borrowed from material provided by CNED (Centre National d’Enseignement à Distance). Texts were adapted and illustrations were added so as to create seven short texts (length between 85 and 227 words), forming a logical progression like in a textbook, and constituting seven modules in Didask. For each of the seven short texts, five or six (depending on the length of the text) multiple choice questions were created for the training phase by selecting five or six main facts from the corresponding text. Thus, all the facts questioned in the training quizzes were covered in the corresponding text; but some facts included in the text passages remained unquestioned. An elaborative feedback was given immediately after each answer, indicating the correctness of the answer and providing explanations directly extracted from the corresponding text passage (feedback explanations did not exceed one or two sentences). A total of 38 multiple choice questions were created for the training session. These multiple choice questions were either single answer questions (*n* = 30) or multiple answers questions (*n* = 8) and we proposed between two and four different choices.

We also created 21 additional questions included in the final exam only, not in the training quizzes. This was to assess the transfer of the retrieval practice effects to untested questions. These untested questions were of two categories: 10 new questions that were directly related to the written information presented in the readings, and 11 generalisation questions that were more difficult, cutting across several learning modules and requiring inferences. Thus, 10 new multiple choice questions were created in the same way as the question included in the training quizzes (information present in the text passages of the learning contents). Furthermore, 11 generalisation questions were also created to measure indirect benefit of the retrieval practice. The answers were not literally included in the learning material, but required inferring or synthesising information across several texts (e.g., placing cell constituents correctly on a picture of a cell that was not included in the learning material). Finally, nine “catch” questions were interspersed throughout the training quizzes and the final test to ensure participants were paying proper attention and were not answering randomly (e.g., What is the colour of Henri IVth’s white horse?), and to be used as exclusion criteria (data from participants who correctly answered fewer than seven of the nine “catch” questions during the training phase were excluded).

The final test therefore included a total of 52 questions: 26 randomly selected from the set of the training phase’s questions (i.e., trained), 10 new, 11 generalisation, and 5 catch questions. Thus, the final test contained the same number of trained and untrained questions, and each module was equivalently represented.

### Design and procedure

In the present study we used a mixed factorial design. This type of design usually includes at least one between subject variable and at least one within subject variable,^34^ which in the case of this study were three between-subject Learning Conditions (quiz-reading, reading-quiz and reading-reading) for the acquisition phase and two within-subject Question Types (trained and untrained) for the final test.

**Fig. 2:**
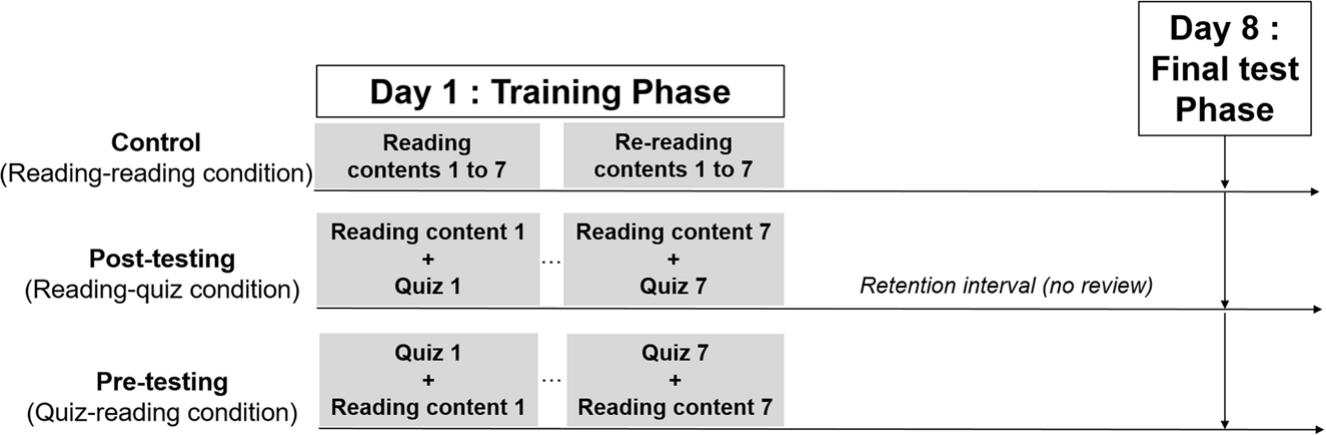
Schema of the experimental procedure used in the three learning conditions

After giving their consent and filling in a demographical questionnaire, participants were allocated alternatingly to one of the three conditions based on order of registration, and provided with an individual link to the testing platform on Didask. All learning and testing took place online, from participants’ personal computers (uncontrolled settings, such as their homes or offices). The experiment consisted in a training phase and a test phase, separated by 7 days (Fig. 2).

#### Training phase

In the quiz-reading condition, clicking on a module first sent participants to the corresponding quiz. No time limit was imposed to answer. After answering each question, feedback immediately appeared and was displayed for at least 10 s, and until they decided to go on to the next question. After completing the last question of the quiz, participants were asked to study the learning content associated to that module, which was displayed for at least 50 s, and until they decided to go on to the next module. This sequence repeated itself from module 1 to 7 (Fig. 2).

In the reading-quiz condition, the procedure was exactly the same except that when clicking a module, the learning content first appeared for at least 50 s, then when clicking to continue the quiz with feedback started. This was repeated from module 1 to 7. These two conditions included exactly the same 38 training multiple choice questions; the only difference was the relative placement of quizzes and reading. For both conditions with testing, the training phase for all seven modules lasted about 30 min in total. No additional testing or reading of the material took place between this training phase and the final test phase.

In the reading-reading condition, only the learning content was presented in each module. The participants had to go from the first to the seventh module to study each content for at least 50 s. Once they had finished the 7th module, they were sent again to the 1st module for a second study phase under the same conditions. This learning condition lasted about 15 min in total. Because durations differed between the three conditions, they were recorded and taken into account for data analysis (i.e. time on training phase did not influence the main effects).

Participants were paid for the training phase only after successful completion of the training procedure. The instructions required the participants to go through each module only once (or twice in the reading-reading condition), however for technical reasons we were not able to block extra uses of each module. We therefore recorded the total number of module visits in order to take them into account for data analysis (see Covariates and exploratory analyses in “Results” section). The learning phase was open to new participants for about 28 h, from the time we published the advertisement to the time we obtained 334 participants and decided to close the task on Foule Factory. The access to the training space on Didask was closed after the last participant completed the learning phase. Participants were instructed not to study more about DNA before the test phase. This was of course impossible to control, but there was no incentive for further study, given that payment was a flat rate for participation, regardless of performance in the final test.

#### Final test phase

Participants who completed the learning phase correctly were asked to participate in the final test phase for an additional payment and 7 days after the learning phase (mean retention interval = 6.91 days (SD = 0.20; range = 6.27–7.59 days)). Questions that were presented in this final test were the same and in the same order for all participants. There was no time limit to complete the final test. It took about 15 min. No feedback was given after any of the 52 questions, but at the end of the test participants were given their total score.

In both learning and test phases, each question was scored 1 if the answer was entirely correct and 0 when any error was made. Percentage correct answers across all seven modules were then analysed.

### Reporting summary

Further information on research design is available in the Nature Research Reporting Summary linked to this article.

## Supplementary information

Reporting Summary

## Data Availability

The preregistration for this experiment can be accessed at http://aspredicted.org/blind.php?x=wg7nj6. An initial version of the preregistration planned final test at both 7 and 28 days post training. However, during the preparation of the experiment, we decided to drop the 28-day test, hence the new version of the preregistration. De-identified data, and materials are posted on Dataverse website with the link:^35^ 10.7910/DVN/XPYPMF.

## Supplementary information

**Supplementary information** accompanies the paper on the *npj Science of Learning* website (10.1038/s41539-019-0053-1).

## Code availability

Data analysis scripts for this experiment along with a code-book are posted on Dataverse website with the following link:^35^ 10.7910/DVN/XPYPMF.
